# Improved vascular depiction and image quality through deep learning reconstruction of CT hepatic arteriography during transcatheter arterial chemoembolization

**DOI:** 10.1007/s11604-024-01614-3

**Published:** 2024-06-18

**Authors:** Yukichi Tanahashi, Koh Kubota, Takayuki Nomura, Takanobu Ikeda, Masaya Kutsuna, Satoshi Funayama, Tatsunori Kobayashi, Kumi Ozaki, Shintaro Ichikawa, Satoshi Goshima

**Affiliations:** 1 Department of Radiology, Hamamatsu University School of Medicine, 1-20-1 Handayama, Chuo-ku, Hamamatsu City, Shizuoka 431-3192 Japan; 2 Radiology Service, Hamamatsu University Hospital, Hamamatsu City, Shizuoka Japan

**Keywords:** CT during hepatic arteriography, Deep learning reconstruction, Transarterial chemoembolization, Hepatic artery

## Abstract

**Purpose:**

To evaluate the effect of deep learning reconstruction (DLR) on vascular depiction, tumor enhancement, and image quality of computed tomography hepatic arteriography (CTHA) images acquired during transcatheter arterial chemoembolization (TACE).

**Methods:**

Institutional review board approval was obtained. Twenty-seven patients (18 men and 9 women, mean age, 75.7 years) who underwent CTHA immediately before TACE were enrolled. All images were reconstructed using three reconstruction algorithms: hybrid-iterative reconstruction (hybrid-IR), DLR with mild strength (DLR-M), and DLR with strong strength (DLR-S). Vascular depiction, tumor enhancement, feeder visualization, and image quality of CTHA were quantitatively and qualitatively assessed by two radiologists and compared between the three reconstruction algorithms.

**Results:**

The mean signal-to-noise ratios (SNR) of sub-segmental arteries and sub-sub-segmental arteries, and the contrast-to-noise ratio (CNR) of tumors, were significantly higher on DLR-S than on DLR-M and hybrid-IR (*P* < 0.001). The mean qualitative score for sharpness of sub-segmental and sub-sub-segmental arteries was significantly better on DLR-S than on DLR-M and hybrid-IR (*P* < 0.001). There was no significant difference in the feeder artery detection rate of automated feeder artery detection software among three reconstruction algorithms (*P* = 0.102). The contrast, continuity, and confidence level of feeder artery detection was significantly better on DLR-S than on DLR-M (*P* = 0.013, 0.005, and 0.001) and hybrid-IR (*P* < 0.001, *P* = 0.002, and *P* < 0.001). The weighted kappa values between two readers for qualitative scores of feeder artery visualization were 0.807–0.874. The mean qualitative scores for sharpness, granulation, and diagnostic acceptability of CTHA were better on DLR-S than on DLR-M and hybrid-IR (*P* < 0.001).

**Conclusions:**

DLR significantly improved the SNR of small hepatic arteries, the CNR of tumor, and feeder artery visualization on CTHA images. DLR-S seems to be better suited to routine CTHA in TACE than does hybrid-IR.

## Introduction

Transcatheter arterial chemoembolization (TACE) is a standard treatment option for intermediate-stage hepatocellular carcinoma (HCC). The indications for TACE for intermediate-stage HCC have changed during the past decade because of marked developments in systemic therapy [1–7]. This paradigm shift in treatment strategy for intermediate-stage HCC has also seen changes in TACE procedures to provide more curative TACE and maintain liver function.

Recently, selective conventional TACE (cTACE) using iodized oil was reported to be superior to selective TACE using drug-eluting beads [8]. Superselective TACE is defined as cTACE at the most distal portion of the sub-sub-segmental hepatic artery, and has therapeutic effects on not only hypervascular tumor portions, but also on hypovascular tumor portions [9]. Although evaluation of small feeding arteries is essential for performing superselective TACE, the sensitivity of digital subtraction angiography (DSA) for detecting tumors and feeding arteries is rather low (67% and 55%) [10].

Previous reports showed the efficacy of computed tomography hepatic arteriography (CTHA) during TACE [10–13], with it providing better visualization of tumor and small feeder arteries than DSA. Recently, the efficacy of automated feeder artery detection software applied to CTHA for TACE was reported [9, 14, 15], and automated feeder artery detection software guidance using CTHA images has become a standard technique for superselective cTACE. Thus, improvement of CTHA images could contribute to more curative TACE.

Deep-learning reconstruction (DLR) uses deep convolutional models based on neural networks. Previous reports showed that DLR reduced image noise and increased the signal-to-noise ratio (SNR) and contrast-to-noise ratio (CNR) [16–19]. We hypothesized that DLR would improve CTHA images acquired during TACE. However, to our knowledge, no study has reported the efficacy of DLR for CT acquired during arteriography. Thus, the goal of this study was to evaluate the influence of DLR on the vascular depiction and image quality of CTHA acquired during TACE, and to assess the optimal strength of DLR for CTHA.

## Materials and methods

### Patients

This retrospective study was approved by our institutional review board (Hamamatsu University School of Medicine), and the requirement for written informed consent was waived. From July 2021 to April 2022, 33 patients underwent CTHA (a total of 38 procedures) for the treatment of hepatic lesions. We excluded six patients because of a lack of raw CTHA image data, and the remaining 27 patients (18 men, 9 women; age [mean ± standard deviation]: 75.7 ± 9.7 years; age range: 53–91 years) were enrolled in this study. For those patients who underwent TACE two or more times, the first session was used in this study. The target lesions for TACE were: HCC (*n* = 24), liver metastasis from rectal cancer (*n* = 1), and liver metastasis from pancreatic neuroendocrine neoplasm (*n* = 2). The indication for TACE was decided at a multidisciplinary meeting. The patients’ background data are shown in Table 1.

**Table 1:** Patients’ background

Case	Age (years)	Sex	BMI (kg / m^2^)	Etiology	Tumor	Number of lesions
1	70	M	22.1	NA	HCC	2
2	85	F	23.1	Hepatitis C	HCC	2
3	77	M	30.5	Hepatitis C	HCC	2
4	76	M	23.6	Hepatitis C	HCC	2
5	66	M	24.2	NA	Metastasis (rectal cancer)	1
6	82	F	24.8	Hepatitis C	HCC	1
7	70	F	35.9	Nonalcoholic	HCC	1
8	75	M	27.1	Alcohol	HCC	1
9	88	M	24.9	Hepatitis C	HCC	1
10	70	F	32.5	Alcohol	HCC	5
11	73	F	22.2	Hepatitis C	HCC	1
12	89	M	25.4	Hepatitis C	HCC	1
13	91	M	18	Hepatitis C	HCC	1
14	59	M	21	Hepatitis B	HCC	1
15	86	M	31.7	Nonalcoholic fatty liver disease	HCC	1
16	79	F	28.2	NA	Metastasis (pancreatic NET)	3
17	53	M	25.6	Hepatitis B	HCC	2
18	65	F	34.3	Hepatitis B	HCC	Multiple
19	90	M	20.5	Hepatitis C	HCC	1
20	68	M	NA	Alcohol	HCC	4
21	80	F	25.4	Hepatitis C	HCC	1
22	65	M	22.6	NA	Metastasis (pancreatic NET)	3
23	78	M	18.7	Hepatitis B	HCC	1
24	86	M	18.3	Hepatitis C	HCC	1
25	76	F	28.8	Hepatitis C	HCC	1
26	71	M	25.8	Hepatitis C	HCC	3
27	76	M	23.1	Hepatitis B	HCC	multiple

*HCC* hepatocellular carcinoma, *NET* neuroendocrine tumor, *NA* not applicable

### CTHA procedure and protocol

The CTHA and TACE procedures were performed using a hybrid angiography CT system. A 5-F preshaped loop catheter (GHC-A; Hanaco medical CO, LTD., Saitama, Japan) was inserted into the common hepatic artery. When a loop catheter was not suitable for the hepatic artery anatomy, a 4-F preshaped shepherd hook catheter (Medikit’s Angiographic Catheter; Medikit CO, LTD., Tokyo, Japan) was used. CTHA was obtained 6, 21.5, and 42 s after the initiation of the contrast media injection (dual injection of normal saline and 300 mgI/mL water-soluble iodine contrast media [Iopamiron 300; Bayer AG, Leverkusen, Germany] in a 1:1 ratio) for volume scans and 6, 23.5, and 41.5 s after for helical scans [20]. The flow rate was determined based on the DSA of the common hepatic artery. The duration of contrast media injection was 26 s for helical scan and 21 s for volume scan mode.

A 320-detector CT scanner (Aquilion ONE/NATURE Edition; Canon Medical Systems Corp., Tochigi, Japan) with automatic tube current modulation (auto exposure control; AEC) was used for CTHA. The tube current modulation program combined z-axis and angular modulation of the X-ray tube current adjusted for the patient’s body size and shape (monitored from a single positioning image) to account for all three dimensions. Using the scout scan and a preset noise index, the system modulates the tube current during CT scanning to achieve an acceptable image noise level. Volume and helical scans were used and selected according to the patient’s liver size.

The following CT imaging parameters were used: tube voltage, 120 kVp; tube current, 50–550 mA (AEC); noise index (SD value), 8.5; detector configuration, 320 detectors with 0.5-mm section thickness for volume scan and 80 detectors with 0.5-mm section thickness for helical scan; beam collimation, 160 mm for volume scan and 40 mm for helical scan; rotation time, 0.5 s; pitch factor, not applicable for volume scan and 0.813 for helical scan; scan field of view (FOV), M or L; display FOV, 32 cm; scan length, 160 mm for volume scan and 200 mm for helical scan; total exposure time, 0.5 s for volume scan and 4 s for helical scan.

All CTHA images were reconstructed using three different image-reconstruction methods: standard adaptive iterative dose reduction 3D (AIDR 3D Mild; Canon Medical Systems Corp.) (here defined as hybrid-IR), and mild- and strong-strength DLR (DLR-M and DLR-S, respectively; Advanced intelligent Clear-IQ Engine [AiCE] Body Sharp Mild and AiCE Body Sharp STR; Canon Medical Systems Corp.).

### Quantitative image analysis

Two radiologists (INITIALS BLINDED, with 10 and 2 years, respectively, of post-training experience in interpreting body CT images and interventional radiology) measured the CT values of sub-segmental artery (A1–A8), sub-sub-segmental artery (A2s–A8s), tumor, liver parenchyma, and subcutaneous fat using 2–10-mm-diameter regions-of-interest (ROIs) on 0.5-mm-thick axial first-phase CTHA images using a 3D workstation (SYNAPSE VINCENT, FUJIFILM Co., Tokyo, Japan). ROIs for artery were placed on CTHA images, encompassing as much of the vascular lumen as possible while avoiding vascular walls, calcification, thrombus, and artifacts. The ROI size for vessel measurement was determined as the maximum size of the vessel without including vessel wall. ROIs for tumor were placed on the strongest enhancement area. ROIs for liver parenchyma were placed so as not to involve the major vessels, tumor, and artifact. When arterial branches were partly lacking because of prior treatment, they were managed as missing values. The sub-sub-segmental branch of A1 was not evaluated in this study because of its small diameter. In patients with multiple tumors, the largest and second largest tumor on which TACE was performed were evaluated. The SNR was calculated by dividing the CT values of artery by the standard deviation (SD) of the CT values of subcutaneous fat. The CNR of tumor was calculated by dividing the difference in CT values between lesion and liver parenchyma by the SD of the CT values of subcutaneous fat. Image noise was defined as the SD of subcutaneous fat.

### Qualitative image analysis

Two radiologists (INITIALS BLINDED, with 10 and 2 years, respectively, of post-training experience in interpreting body CT images and interventional radiology), who were unaware of the reconstruction methods, reviewed the first-phase CTHA images using a 3D workstation (SYNAPSE VINCENT). CTHA images were initially presented with a preset soft-tissue window setting (320 HU width and 120 HU level), with the radiologists being allowed to modify the window setting at their own discretion.

The radiologists graded vascular depiction (sharpness and contrast), lesion contrast, and image quality (sharpness, granulation, artifact, and overall diagnostic availability) using a five-point rating scale: 5 for excellent, 4 for good, 3 for acceptable, 2 for suboptimal, and 1 for unacceptable. To minimize learning bias, each review for hybrid-IR, DLR-M, and DLR-S was performed with a time interval of at least 2 weeks.

### Feeder artery analysis

A radiologist (INITIALS BLINDED, with 10 years of post-training experience in interpreting body CT images and interventional radiology) retrospectively reviewed the angiography and identified the tumor-feeder arteries. This radiologist also retrospectively applied automated feeder artery detection software (Embolization Plan; Canon Medical Systems Corp.) to the CTHA images and evaluated its detection rate for feeder arteries. The volume of interest (VOI) for the automated feeder artery detection software was manually drawn by the radiologist on the target lesion and peritumoral area. Partial mistracing of the route of a feeder artery by the software was counted as a failure.

Two radiologists (INITIALS BLINDED, with 2 years of post-training experience in interpreting body CT images and interventional radiology) who were unaware of the tumor-feeder artery independently reviewed the CTHA images and evaluated the number of tumor-feeder arteries and tumor-feeder artery visualization (contrast, continuity, and confidence level) using a 4-point rating scale, and then reviewed the evaluations in consensus. The reviewers scored feeder artery contrast according to a 4-point rating scale: 4 for excellent, 3 for good, 2 for fair, and 1 for bad. The reviewers scored feeder artery continuity using 4-point rating scale: 4 for no discontinuities, 3 for some discontinuity but less than 50%, 2 for around 50% discontinuity, and 1 for more than 50% subject to discontinuity. The reviewers also scored feeder artery confidence level using 4-point rating scale: 4 for 100% of confidence level, 3 for more than 50% but less than 100% of confidence level, 2 for around 50% of confidence level, and 1 for less than 50% of confidence level.

### Statistical analysis

Statistical analyses were performed using SPSS for Windows (version 26.0; IBM Corp., Armonk, NY, USA). Quantitative values were compared between the three different reconstruction methods (hybrid-IR, DLR-M, and DLR-S) using repeated-measures analysis of variance and post hoc Tukey tests. Qualitative scores were compared using the Friedman test followed by pair-wise comparisons using the Wilcoxon signed-rank test. Cochran’s *Q* test was used for the analysis of feeder artery detection rate. *P* values less than 0.05 were considered statistically significant. The Bonferroni correction adopting a stricter *P* value of less than 0.017 was used for pair-wise comparisons.

Weighted kappa analysis was conducted to assess interobserver variability in the evaluation of image quality and feeder artery visualization. A kappa value of up to 0.20 was considered to indicate slight agreement; 0.21–0.40, fair agreement; 0.41–0.60, moderate agreement; 0.61–0.80, substantial agreement; and 0.81 or greater, almost perfect agreement.

## Results

**Fig. 1:**
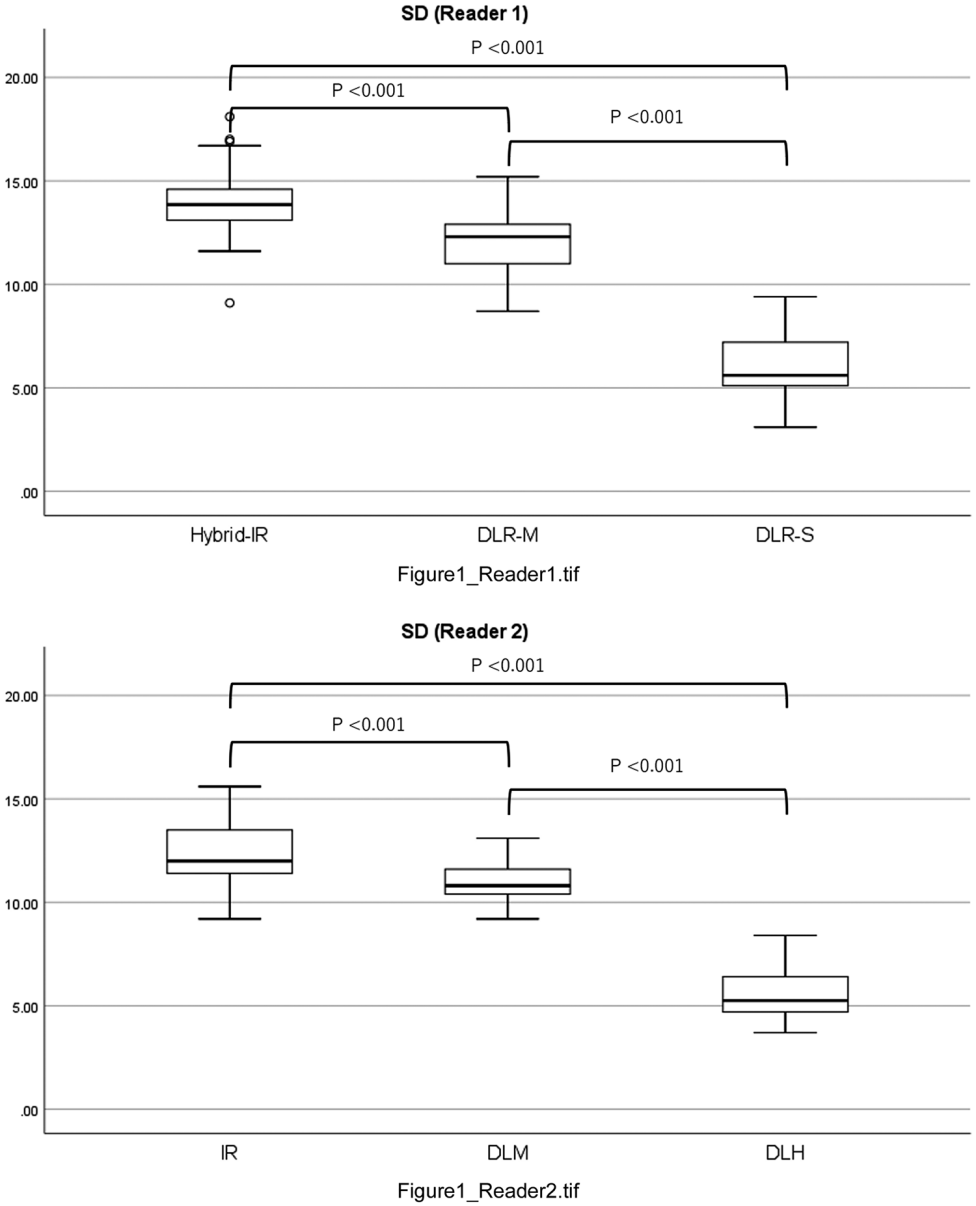
The mean standard deviation (SD) of subcutaneous fat. For both readers, the SD of subcutaneous fat was significantly lower on DLR-S than on DLR-M and hybrid-IR. *IR* iterative reconstruction, *DLR-M* deep learning reconstruction with mild strength, *DLR-S* deep learning reconstruction with strong strength

The mean numbers of sub-segmental arteries and sub-sub-segmental arteries were 22 and 19, respectively. The mean CT values of sub-segmental and sub-sub-segmental arteries were significantly higher on DLR-S (795.4 HU and 720.2 HU, respectively, for reader 1; 838.6 HU and 724.8 HU for reader 2) and DLR-M (777.0 HU and 705.0 HU for reader 1; 822.0 HU and 702.4 HU for reader 2) than on hybrid-IR (624.8 HU and 558.8 HU for reader 1; 652.7 HU and 555.0 HU for reader 2) (both *P* < 0.001; Table 2). The mean SD in subcutaneous fat was significantly lower on DLR-S (6.0 for reader 1; 5.6 for reader 2) than on DLR-M (12.0 for reader 1; 11.0 for reader 2) and hybrid-IR (13.9 for reader 1; 12.3 for reader 2) (all *P* < 0.001; Fig. 1). The mean SNRs of sub-segmental and sub-sub-segmental arteries, and the CNR of tumors, were significantly higher on DLR-S (139.3, 124.9, and 27.1, respectively, for reader 1; 159.3, 136.7, and 32.2 for reader 2) than on DLR-M (65.8, 59.6, and 13.1 for reader 1; 75.2, 64.3, and 15.3 for reader 2) and hybrid-IR (45.4, 40.6, and 10.8 for reader 1; 53.8, 45.7, and 13.2 for reader 2) (*P* < 0.001 for all; Table 3).

**Table 2:** CT value of hepatic arteries

	Hybrid-IR	DLR-M	DLR-S	*P* value (DLR-S vs DLR-M)	*P* value (DLR-S vs hybrid-IR)	*P* value (DLR-M vs hybrid-IR)
Sub-segmental artery						
Reader 1	624.8 ± 317.8^a,b^	777 ± 388^b^	795.4 ± 398.9^a^	0.722	< 0.001	< 0.001
Reader 2	652.7 ± 334.4^a,b^	822 ± 425.3^b^	838.6 ± 442.3^a^	0.819	< 0.001	< 0.001
Sub-sub-segmental artery						
Reader 1	558.8 ± 251.7^a,b^	705 ± 329^b^	720.2 ± 340.8^a^	0.743	< 0.001	< 0.001
Reader 2	555 ± 246.9^a,b^	702.4 ± 347.9^b^	724.8 ± 361.4^a^	0.616	< 0.001	< 0.001

Unless otherwise indicated, data are means ± standard deviation

*CT* computed tomography, *IR* iterative reconstruction, *DLR-M* deep learning reconstruction with mild strength, *DLR-S* deep learning reconstruction with strong strength

^*^Significant difference between DLR-M and DLR-S

^a^Significant difference between hybrid-IR and DLR-S

^b^Significant difference between hybrid-IR and DLR-M

**Table 3:** SNR of hepatic arteries and CNR of lesion

	Hybrid-IR	DLR-M	DLR-S	*P* value (DLR-S vs DLR-M)	*P* value (DLR-S vs hybrid-IR)	*P* value (DLR-M vs hybrid-IR)
Sub-segmental artery						
Reader 1	45.4 ± 22.3^a,b^	65.8 ± 32.9^*,b^	139.3 ± 73.3^*,a^	< 0.001	< 0.001	< 0.001
Reader 2	53.8 ± 27.1^a,b^	75.2 ± 38.4^*,b^	159.3 ± 94.9^*,a^	< 0.001	< 0.001	< 0.001
Sub-sub-segmental artery						
Reader 1	40.6 ± 17.6^a,b^	59.6 ± 28.1^*,b^	124.9 ± 61.6^*,a^	< 0.001	< 0.001	< 0.001
Reader 2	45.7 ± 20^a,b^	64.3 ± 31.3^*,b^	136.7 ± 76.2^*,a^	< 0.001	< 0.001	< 0.001
Tumor						
Reader 1	10.8 ± 6.4^a^	13.1 ± 8.4^*^	27.1 ± 17.9^*,a^	< 0.001	< 0.001	0.403
Reader 2	13.2 ± 7.8^a^	15.3 ± 9.5^*^	32.2 ± 21.5^*,a^	< 0.001	< 0.001	0.601

Unless otherwise indicated, data are means ± standard deviation

*SNR* signal-to-noise ratio, *CNR* contrast-to-noise ratio, *IR* iterative reconstruction, *DLR-M* deep learning reconstruction with mild strength, *DLR-S* deep learning reconstruction with strong strength

*Significant difference between DLR-M and DLR-S

^a^Significant difference between hybrid-IR and DLR-S

^b^Significant difference between hybrid-IR and DLR-M

**Fig. 2:**
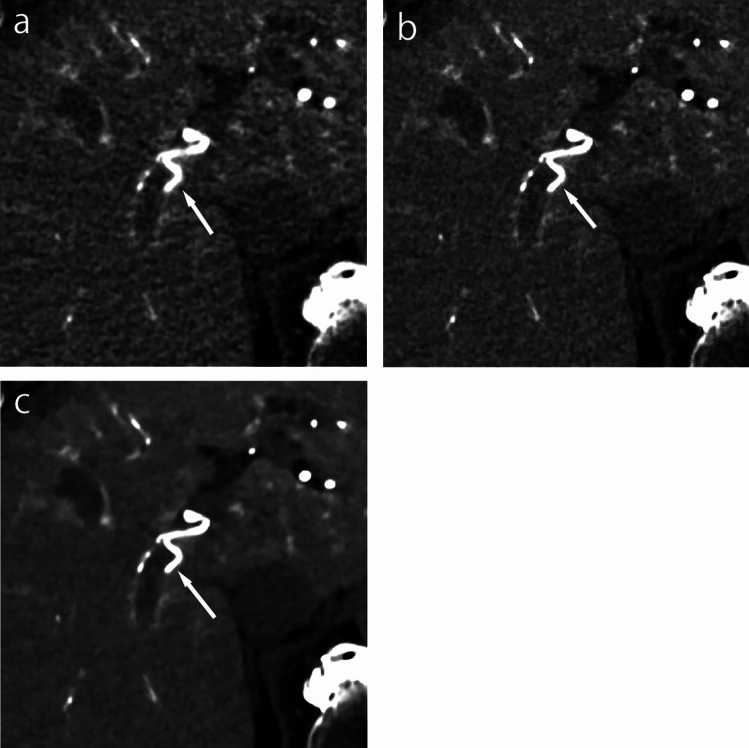
CTHA images with DLR-S, DLR-M, and hybrid-IR. The sharpness and contrast of hepatic arteries (A7: arrows) are better on DLR-S (**c**) than on DLR-M (**b**) and hybrid-IR (**a**). *CTHA* computed tomography during hepatic arteriography, *DLR-S* deep learning reconstruction with strong strength, *DLR-M* deep learning reconstruction with mild strength

The mean qualitative scores for the sharpness and contrast of sub-segmental and sub-sub-segmental arteries are summarized in Table 4. The mean qualitative scores for the sharpness of sub-segmental and sub-sub-segmental arteries were significantly better on DLR-S (4.8 and 4.5, respectively, for reader 1; 4.4 and 4.3 for reader 2) than on DLR-M (3.8 and 3.5 for reader 1; 3.7 and 3.5 for reader 2) and hybrid-IR (2.8 and 2.7 for reader 1; 2.8 and 2.6 for reader 2) (all *P* < 0.001). The mean qualitative scores for contrast of sub-segmental and sub-sub-segmental arteries were significantly better on DLR-S (4.6 and 4.3 for reader 1; 4.5 and 4.2 for reader 2) than on DLR-M (4.2 and 3.9 for reader 1; 4.1 and 3.7 for reader 2) and hybrid-IR (3.2 and 2.8 for reader 1; 3.3 and 2.8 for reader 2) (all *P* < 0.001; Fig. 2).

**Table 4:** Qualitative score for sharpness and contrast of hepatic arteries

	Hybrid-IR	DLR-M	DLR-S	*P* value (DLR-S vs DLR-M)	*P* value (DLR-S vs hybrid-IR)	*P* value (DLR-M vs hybrid-IR)
Sharpness						
Sub-segmental artery						
Reader 1	2.8 ± 0.4^a,b^	3.8 ± 0.6^*,b^	4.8 ± 0.5^*,a^	< 0.001	< 0.001	< 0.001
Reader 2	2.8 ± 0.4^a,b^	3.7 ± 0.6^*,b^	4.4 ± 0.8^*,a^	< 0.001	< 0.001	< 0.001
Sub-sub-segmental artery						
Reader 1	2.7 ± 0.5^a,b^	3.5 ± 0.7^*,b^	4.5 ± 0.8^*,a^	< 0.001	< 0.001	< 0.001
Reader 2	2.6 ± 0.5^a,b^	3.5 ± 0.6^*,b^	4.3 ± 0.8^*,a^	< 0.001	< 0.001	< 0.001
Contrast						
Sub-segmental artery						
Reader 1	3.2 ± 0.8^a,b^	4.2 ± 1.0^*,b^	4.6 ± 0.7^*,a^	< 0.001	< 0.001	< 0.001
Reader 2	3.3 ± 0.8^a,b^	4.1 ± 0.9^*,b^	4.5 ± 0.7^*,a^	< 0.001	< 0.001	< 0.001
Sub-sub-segmental artery						
Reader 1	2.8 ± 0.7^a,b^	3.9 ± 1.0^*,b^	4.3 ± 0.9^*,a^	< 0.001	< 0.001	< 0.001
Reader 2	2.8 ± 0.7^a,b^	3.7 ± 0.8^*,b^	4.2 ± 0.8^*,a^	< 0.001	< 0.001	< 0.001

Unless otherwise indicated, data are means ± standard deviation

*SNR* signal-to-noise ratio, *IR* iterative reconstruction, *DLR-M* deep learning reconstruction with mild strength, *DLR-S* deep learning reconstruction with strong strength

^*^Significant difference between DLR-M and DLR-S

^a^Significant difference between hybrid-IR and DLR-S

^b^Significant difference between hybrid-IR and DLR-M

For reader 1, the qualitative score for lesion contrast was significantly better on DLR-S (4.4) than on DLR-M (3.6) and hybrid-IR (3.4) (*P* < 0.001). For reader 2, there was no significant difference in the qualitative score for lesion contrast (*P* = 0.046 [Bonferroni corrected significance level = 0.017] for DLR-S vs DLR-M, *P* = 0.086 for DLR-S vs hybrid-IR, *P* = 0.949 for DLR-S vs hybrid-IR).

**Fig. 3:**
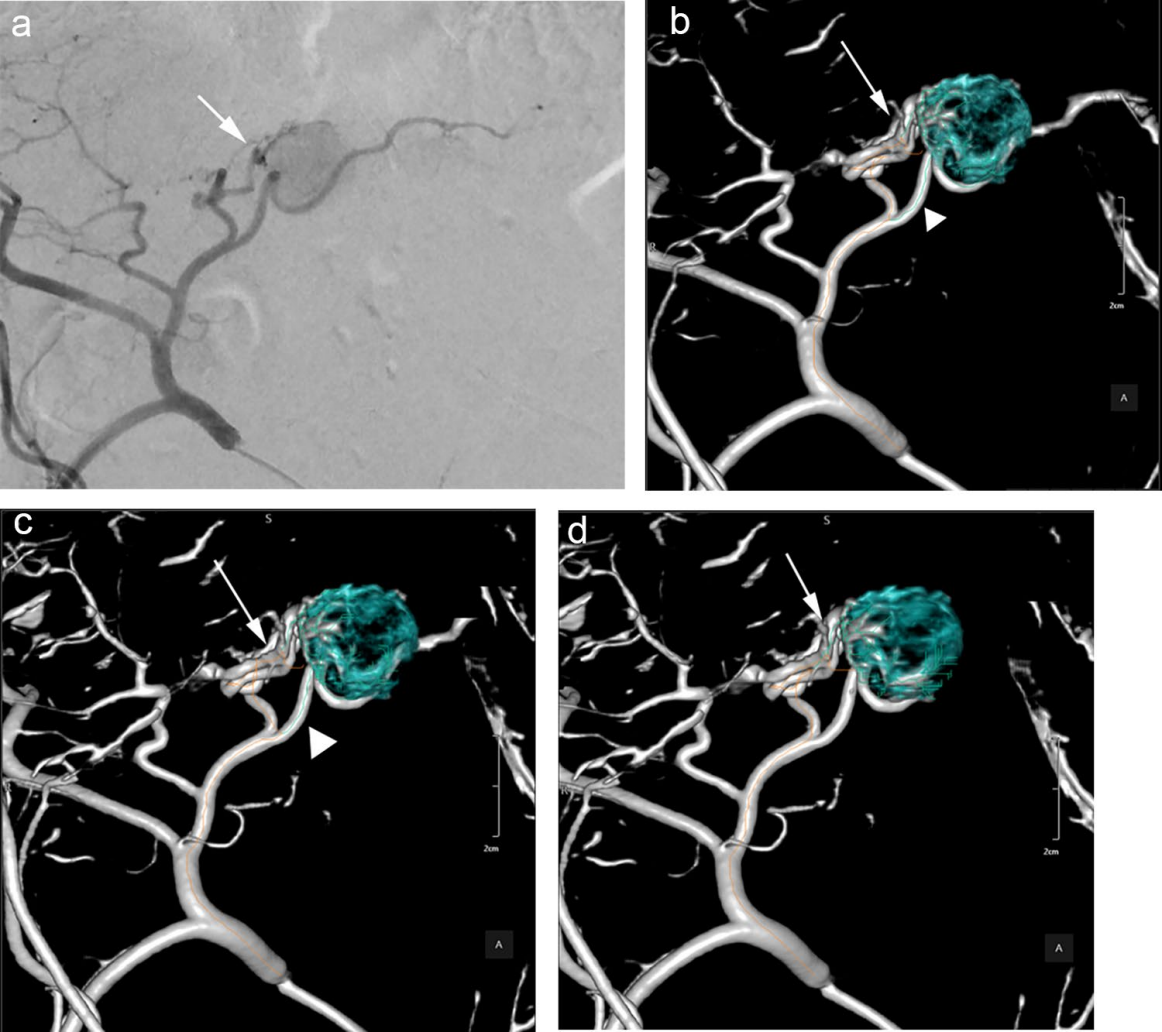
The images of automated tumor-feeder artery detection software using DLR-S. DLR-M, and hybrid-IR images. 53 years old male was performed selective TACE for HCC in segment S2. Angiography (**a**) revealed that the branches of A2 were tumor-feeder arteries (arrow). The image of automated tumor-feeder artery detection software using DLR-S images (**d**) correctly detected feeder artery (arrow). On the other hand, the images of automated tumor-feeder artery detection software using Hybrid-IR images (**b**) and DLR-M images (**c**) showed a false-positive feeder artery (A3; arrowhead), because A3 runs the near to the lesion

The total number of feeder arteries identified on angiography was 47. The detection rates of the automated feeder artery detection software were 70.2% (33/47) on hybrid-IR, 78.7% (37/47) on DLR-M, and 78.7% (37/47) on DLR-S, respectively (*P* = 0.102) (Fig. 3). There were eight false-positive vessels on hybrid-IR, ten on DLR-M, and seven on DLR-S (*P* = 0.374).

**Fig. 4:**
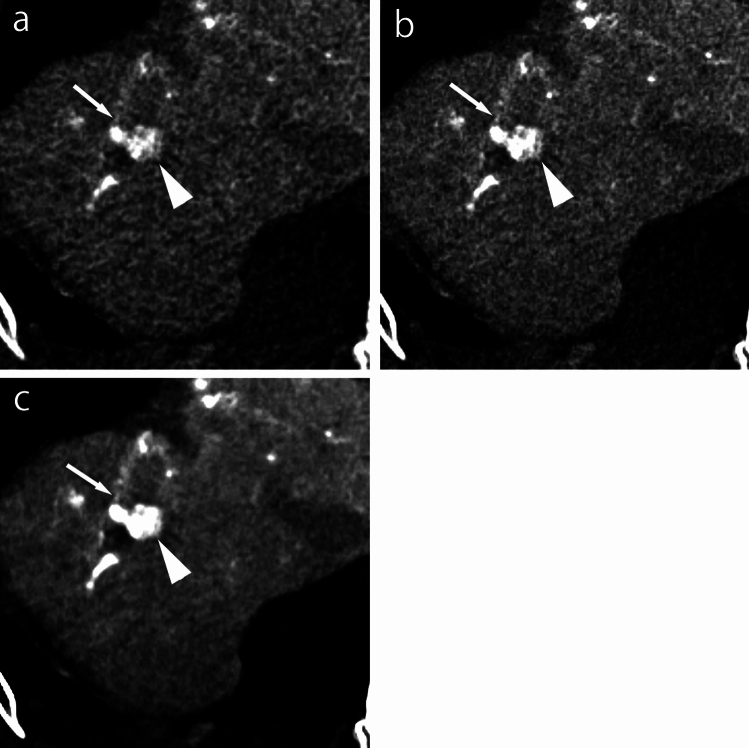
CTHA images with DLR-S, DLR-M, and hybrid-IR. The small feeding artery (arrows) to a hepatic carcinoma (arrow head) is hard to distinguish from image noise on hybrid-IR (**a**) and DLR-M (**b**), but is more distinguishable from image noise on DLR-S (**c**). The tumor-to-liver contrast is better on DLR-S (**c**) than on hybrid-IR (**a**) and DLR-M (**b**). *CTHA* computed tomography during hepatic arteriography, *DLR-S* deep learning reconstruction with strong strength, *DLR-M* deep learning reconstruction with mild strength

The manual assessment feeder detection rates were 85.1% (40/47) for hybrid-IR, 85.1% (40/47) for DLR-M, and 87.2% (41/47) for DLR-S (*P* = 0.368). There were three false-positive vessels on hybrid-IR, three on DLR-M, and two on DLR-S (*P* = 0.375). The mean qualitative scores for feeder artery contrast, continuity, and confidence level were significantly better on DLR-S (3.8, 3.3, and 3.4, respectively) than on DLR-M (3.5, 3.0, and 3.0) and hybrid-IR (3.0, 2.9, and 2.9) (*P* from less than 0.001–0.013; Table 5, Fig. 4). The weighted kappa values between the two readers were 0.866 (0.804–0.928) for feeder artery contrast, 0.807 (0.717–0.897) for feeder artery continuity, and 0.874 (0.803–0.946) for feeder artery confidence level.

**Table 5:** Qualitative score for feeder visualization

Feeder	Hybrid-IR	DLR-M	DLR-S	*P* value (DLR-S vs DLR-M)	*P* value (DLR-S vs hybrid-IR)	*P* value (DLR-M vs hybrid-IR)
Contrast	3.0 ± 1.2^ab^	3.5 ± 1.2^*b^	3.8 ± 1^*a^	0.013	< 0.001	< 0.001
Continuity	2.9 ± 0.9 ^a^	3.0 ± 0.9^*^	3.3 ± 0.8^*a^	0.005	0.002	0.317
Confidence level	2.9 ± 1.0 ^a^	3.0 ± 0.8^*^	3.4 ± 0.7^*a^	0.001	< 0.001	0.102

Unless otherwise indicated, data are means ± standard deviation

*IR* iterative reconstruction, *DLR-M* deep learning reconstruction with mild strength, *DLR-S* deep learning reconstruction with strong strength

^*^Significant difference between DLR-M and DLR-S

^a^Significant difference between hybrid-IR and DLR-S

^b^Significant difference between hybrid-IR and DLR-M

The mean qualitative scores for sharpness, granulation, artifact, and diagnostic acceptability of CTHA were significantly better on DLR-S (4.8, 4.7, 4.4 and 4.8, respectively, for reader1; 4.9, 4.7, 4.3, and 4.8 for reader 2) than on DLR-M (3.8, 3.9, 3.9, and 4.0 for reader 1; 3.9, 3.9, 3.7, and 3.9 for reader 2) and hybrid-IR (3.0, 2.7, 3.7, and 3.1 for reader 1; 3.1, 2.8, 3.3, and 3.1 for reader 2) (*P* ≦ 0.001 for all). The weighted kappa values between the two readers for qualitative scores of sharpness, granulation, artifact, and diagnostic acceptability on CTHA were 0.831 (0.742–0.920), 0.800 (0.714–0.885), 0.412 (0.249–0.575), and 0.970 (0.929–1.011), respectively.

The mean CT dose index volume (mGy) and dose–length product (mGy.cm) were 11.3 and 241.9, respectively, for helical scan, and 7.3 and 116.4 for volume scan.

## Discussion

Our results for both quantitative and qualitative analysis demonstrate that DLR improved the visualization of sub-segmental and sub-sub-segmental arteries, feeder artery, and tumor on CTHA during TACE. In addition, our results also indicate that the higher of the two reconstruction strengths is suitable for CTHA acquired during TACE.

AiCE (Canon Medical Systems Corp.) is one of deep learning-based image-reconstruction methods. AiCE is trained to differentiate signal from noise through training on high-quality patient image datasets acquired with high tube current and reconstructed with model-based iterative reconstruction (MBIR). MBIR images show high spatial resolution and low image noise [21, 22], and therefore, AiCE also results in low image noise, good edge preservation, and good image detail preservation. In this study, DLR significantly decreased the SD of CT values in subcutaneous fat tissue and improved the qualitative scores for image granulation and artifact, indicating that DLR improved image noise. Previous studies that investigated the utility of DLR for abdominal CT also found that DLR significantly decreased image noise, results that are in accord with ours [23, 24]. We also found that DLR significantly increased the CT values of hepatic arteries, which is in accord with a previous report on dynamic abdominal CT that found CT values to be increased with DLR [25]. The decrease in image noise and increase in CT values resulted in an increase in the SNR of hepatic arteries and the CNR of lesion. The improvements in qualitative scores for the sharpness and contrast of hepatic arteries are compatible with these quantitative results. Since DLR itself does not increase CT values directly, we speculate that DLR maintains the “true” CT values by reducing the image noise that may decrease CT values.

In our results, the mean CT values of sub-segmental artery and sub-sub-segmental artery on hybrid-IR images were 624.8 HU and 558.8 HU, respectively, for reader 1, and 652.7 HU and 555.0 HU for reader 2. A previous report on transvenous contrast-enhanced dynamic abdominal CT found that mean CT values in aorta, segmental renal artery, and gastroduodenal artery of 384.3, 219.6, and 102.4 HU, respectively [25]. Transarterial contrast-enhanced CT shows much higher CT values than transvenous contrast-enhanced CT; however, our results show that DLR is feasible for not only transvenous contrast-enhanced CT, but also transarterial contrast-enhanced CT.

We found that in comparison with DLR-M and hybrid-IR, DLR-S reconstruction showed higher SNR in hepatic arteries, higher CNR in lesions, and higher qualitative scores for vascular depiction (sharpness and contrast) and image quality. Since the mean CT values of sub-segmental artery and sub-sub-segmental artery were comparable, we believe that the improvement in the SNR and CNR with the DLR-S in comparison with DLR-M is due to reduced image noise, which is consistent with a previous report [26]. Kaga et al. reported that the conspicuity of hepatic lesions was highest when using iterative reconstruction and tended to lessen as the reconstruction strength level of DLR increased [19]. However, since contrast enhancement is stronger on CTHA than on transvenous contrast-enhanced CT, DLR with higher reconstruction strength would not significantly reduce the conspicuity of hepatic lesions on CTHA. In addition, the better CNR would be beneficial for evaluating tumor vascularity. Thus, DLR with higher reconstruction strength appears suitable for CTHA during TACE. The qualitative score for tumor contrast was better on DLR-S than on DLR-M and hybrid-IR, which is consistent with a previous report that showed that DLR improved HCC detection on abdominal CT [24].

A number of reports have investigated the efficacy of CTHA during TACE. Iwazawa et al. reported that C-arm CT showed a significantly larger area under the receiver-operating characteristics curve than DSA (C-arm CT, Az = 0.995; DSA, Az = 0.841) in the detection of tumor-feeding arteries [11]. Miyayama et al. also investigated the efficacy of cone-beam CT during TACE [12, 13], reporting that the detectability of tumors and tumor-feeding branches with manual feeder vessel detection was significantly better on CT than on nonselective DSA (both *P* < 0.001) [12]. They also reported that the detection rate of automatic feeder vessel detection software was 88% [12]. Because of these advantages, the acquisition of CT during TACE and performing TACE under the guidance of feeder artery detection software have become standard procedures. Thus, improvements in image quality and vessel visualization on CTHA should contribute to improving automatic feeder artery detection rates and the tracing of artery routes, thereby contributing to better clinical outcomes. Our qualitative analysis of feeder visualization revealed that the qualitative scores for feeder artery contrast, continuity, and confidence level were better with DLR-S than with DLR-M and hybrid-IR, although the manual detection rate for tumor-feeder arteries was comparable across the three reconstruction methods. Our results showed that the number of detected feeder artery with automated feeder artery detection software was bigger on DLR-M (37/47) and DLR-S (37/47) than hybrid-IR (33/47). However, it was not statistically significant (*P* = 0.102). We speculate that this might be because the number of cases was small. Further investigation regarding the effect of DLR on the feeder artery detection rate with bigger study population would be required. Iwazawa et al. and Miyayama et al. reported detection rates for automated feeder detection software of 87.7% and 89.6%, respectively [9, 15], while a systematic review reported detection rates of automatic tumor-feeder detection software ranging from 86 to 98.5% [14], values that were higher than those found in the current study. There are three possible reasons for this result. First, our criteria for true positives were strict. In this study, when the software partly mistraced the vessel route, it was counted as a failure. Second, the population was small. Third, the previous studies generally used C-arm CT images, whereas in this study, we used a hybrid angiography CT system. For CTHA, the advantages of hybrid angiography CT system over C-arm CT are better workflow, higher contrast resolution in the low-contrast organs such as soft tissue, and better image quality in obese patients [27, 28].

Radiation dose reduction is another important topic for interventional radiology. Many reports have shown that DLR is efficacious for reducing the radiation dose from abdominal CT [29–32]. Our data indicate that it would be possible to reduce the radiation dose using DLR. However, there is a trade-off between radiation dose and image quality; thus, further investigation is needed to determine the appropriate balance of radiation dose, vessel and tumor visualization, and image quality.

Our study has a number of limitations. First, the number of cases is relatively small. Second, the helical scans and volume scans were not analyzed separately. However, we believe that the difference in scan mode did not have an important effect on the results, because the CT scan parameters’ influence on the CT values and image noise was almost the same on helical and volume scans. Third, this retrospective study did not evaluate clinical outcomes. Fourth, the population included various liver tumors (HCC and liver metastases).

## Conclusions

DLR significantly improved the SNR of small hepatic arteries, the CNR of tumor, and the visualization of feeder artery on CTHA images. DLR-S seemed to be better suited to routine CTHA during TACE than did hybrid-IR.
